# Supplementary material for: Combinatorial mutagenesis of rapidly evolving residues yields super-restrictor antiviral proteins
Source: PLoS Biol. 2019 Oct 1;17(10):e3000181. doi: 10.1371/journal.pbio.3000181 (PMC6772013; doi:10.1371/journal.pbio.3000181)
Supplement: S1 Raw Images — (PDF) [file pbio.3000181.s009.pdf]

Figure 2B
Western blots were incubated with anti-beta actin and anti-FLAG MxA antibodies overnight. Pictures were taken using a ChemiDoc Gel Imaging System adjusting for faint bands. The loading control bands were very faint even in this setting. To obtain a better imagine of the loading control, the blot was stripped using Thermo Scientific's Restore™ Western Blot Stripping Buffer as indicated by the manufacturer. The blots were then washed and incubated with anti-beta actin antibody only overnight followed by secondary HRP-conjugated antibody for 1 hour. After incubation with the substrate, images were re-taken adjusting for faint bands. The resulting images show the beta-actin bands and faint residual MxA bands.

Figure 3A, 3B
Western blots were imaged using the Odyssey® Fc detection system from LI-COR. The secondary antibodies from LI-COR were labeled with immunofluorescence dyes (IRDye® 800CW Goat anti-Rabbit IgG Secondary Antibody (product number: 926-32211); IRDye® 800CW Goat anti-Mouse IgG Secondary Antibody (product number: 926-32210); IRDye® 680LT Goat anti-Rabbit IgG Secondary Antibody (product number: 926-68021)).

Figure 4A
Western blots were incubated with anti-beta actin and anti-FLAG MxA antibodies overnight. The images show a strong signal for FLAG-MxA, but very faint loading band. The blot was cut in between the two lines and the loading controls were re-imaged.

Figures 4B, 5A, 5B
Western blots were incubated with anti-tubulin and anti-FLAG MxA antibodies overnight. Pictures were taken using a ChemiDoc Gel Imaging System adjusting for faint bands.

Figure 4C, 4D
Western blots were incubated with anti-beta actin and anti-FLAG MxA antibodies overnight. Pictures were taken using a ChemiDoc Gel Imaging System adjusting for faint bands.

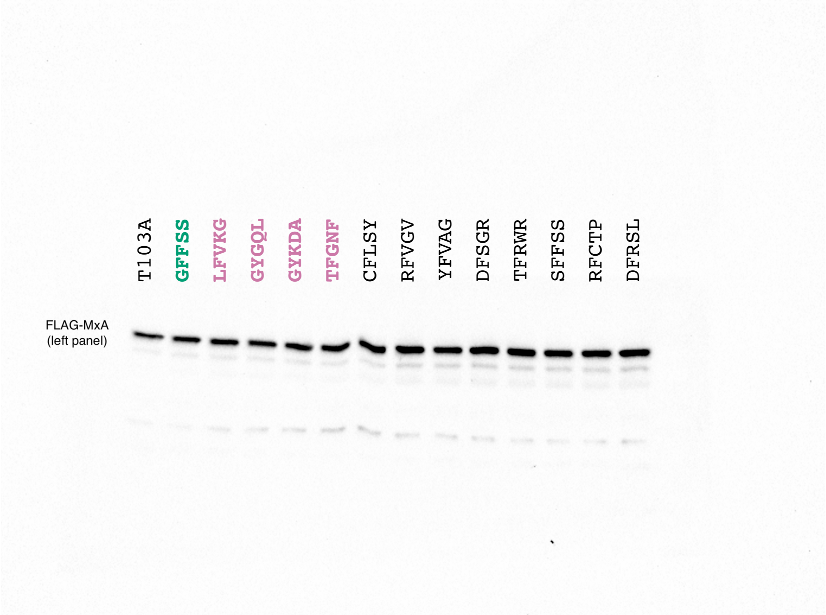
T103A
GFFSS
LFVKG
GYGQL
GYKDA
TFGNF
CFLSY
RFVGV
YFVAG
DFSGR
TFRWR
SFFSS
RFCTP
DFRSL
FLAG-MxA
(left panel)

Fig 2B

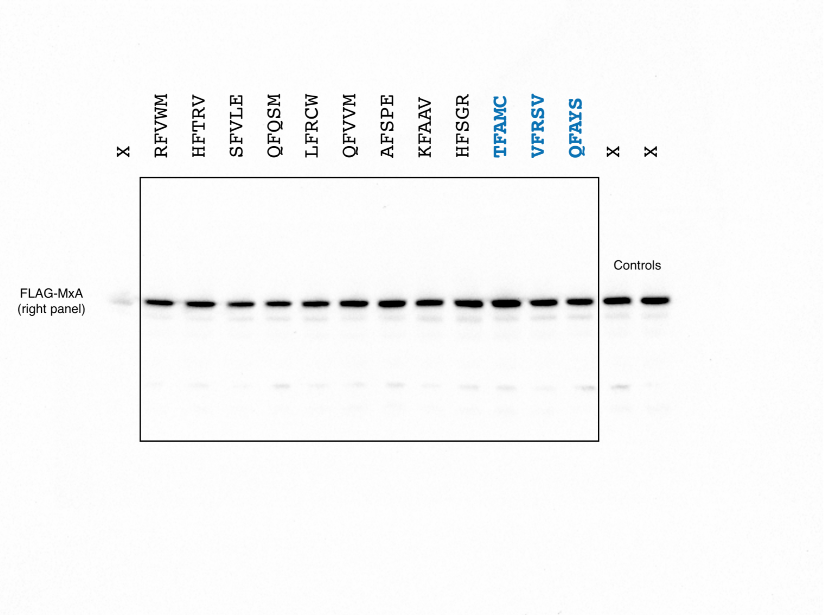

X
RFVWM
HFTRV
SFVLE
QFQSM
LFRCW
QFVVM
AFSPE
KFAAV
HFSGR
TFAMC
VFRSV
QFAYS
X
X
Controls
FLAG-MxA
(right panel)

Fig 2B

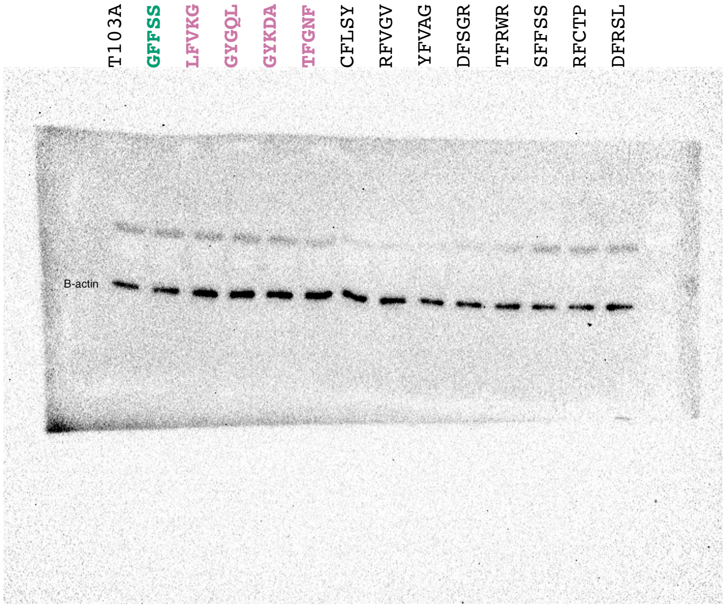
T103A
GFFSS
LFVKG
GYGQL
GYKDA
TFGNF
CFLSY
RFVGV
YFVAG
DFSGR
TFRWR
SFFSS
RFCTP
DFRSL
B-actin

Fig 2B

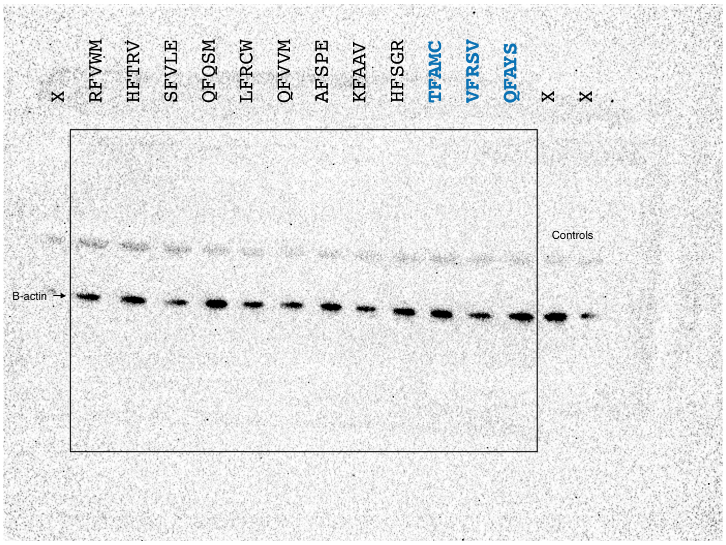
X
RFVWM
HFTRV
SFVLE
QFQSM
LFRCW
QFVVM
AFSPE
KFAAV
HFSGR
TFAMC
VFRSV
QFAYS
X
X
Controls
B-actin

Fig 2B

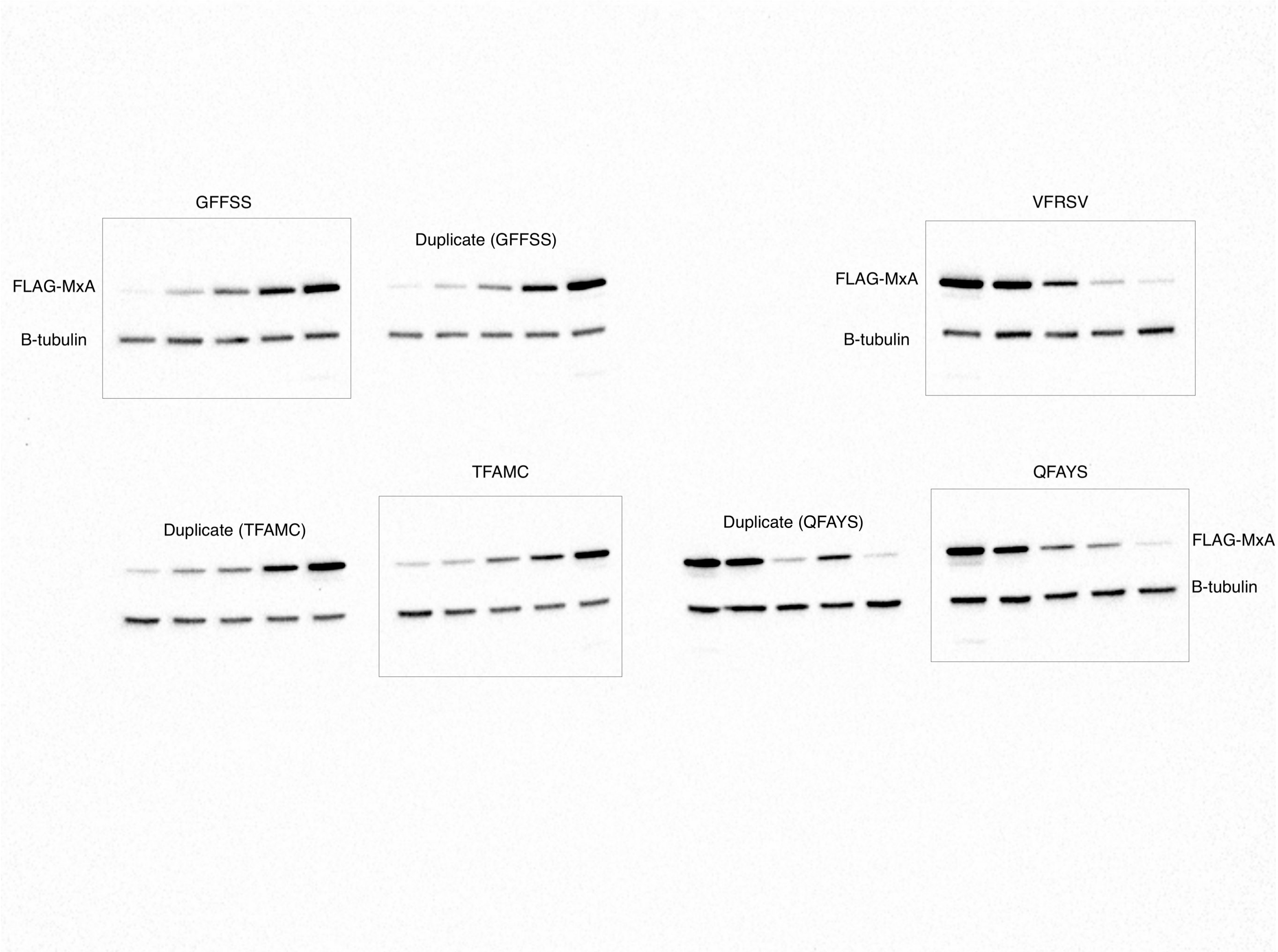
GFFSS
Duplicate (GFFSS)
VFRSV
FLAG-MxA
B-tubulin
FLAG-MxA
B-tubulin
TFAMC
QFAYS
Duplicate (TFAMC)
Duplicate (QFAYS)
FLAG-MxA
B-tubulin

Fig 2C

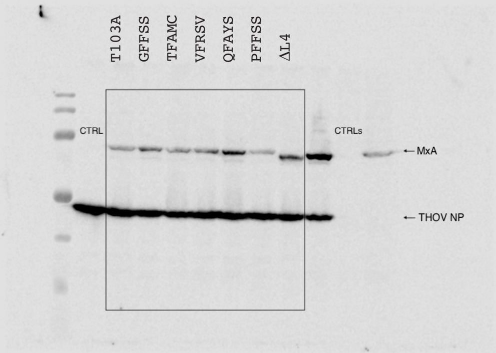
T103A
GFFSS
TFAMC
VFRSV
QFAYS
PFFSS
ΔL4
CTRL
CTRLs
MxA
THOV NP

Fig 3A

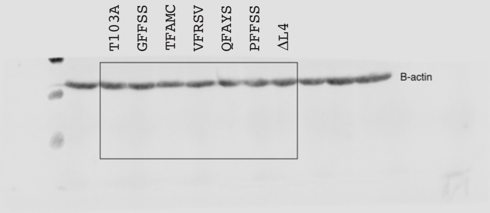

Fig 3A

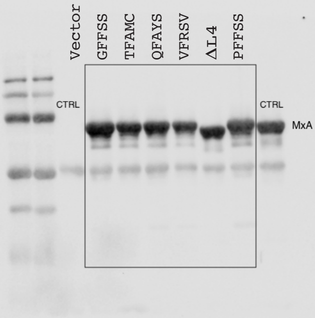

Fig 3B (top row)

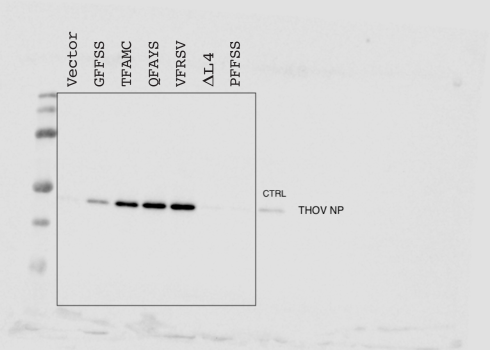

Fig 3B (2nd row)

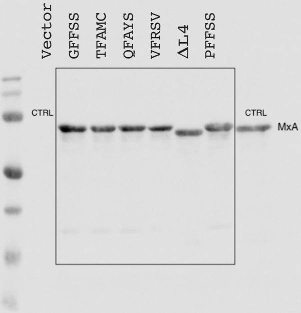

Fig 3B (3rd row)

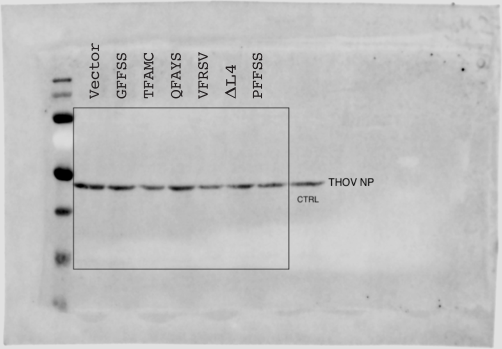

Fig 3B (4th row)

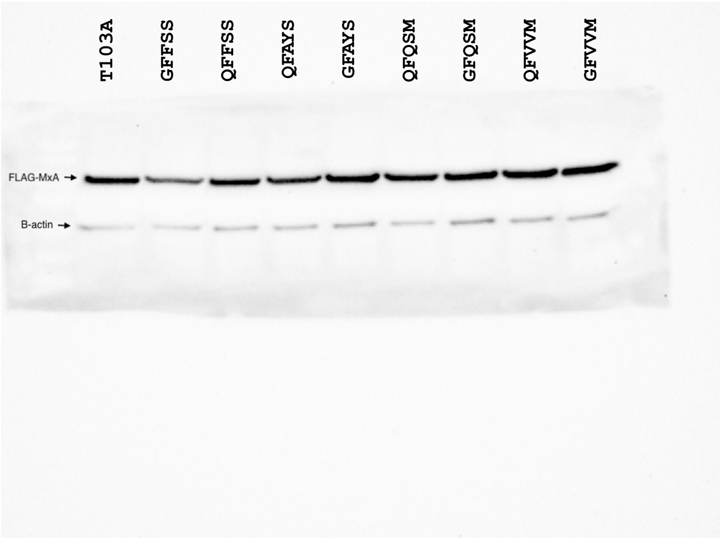

Fig 4A (top row)

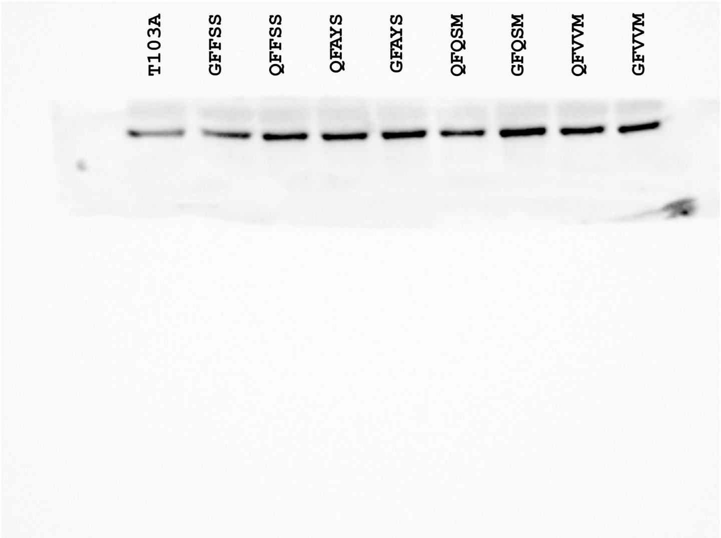

Fig 4A (bottom row)

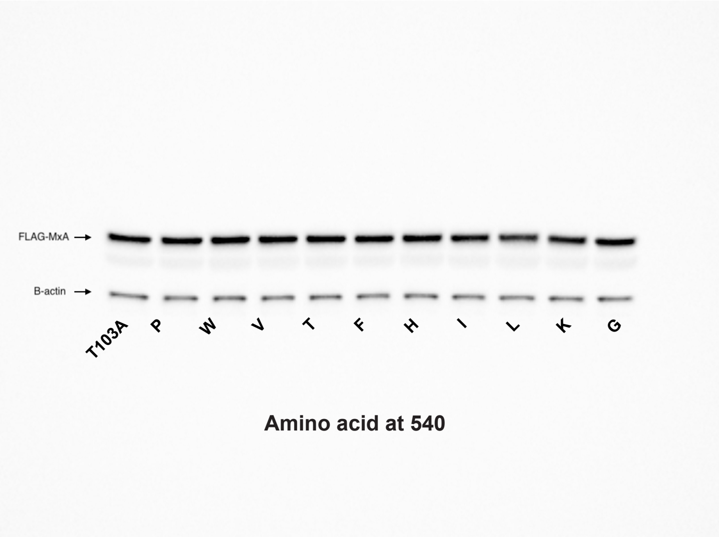

Fig 4B

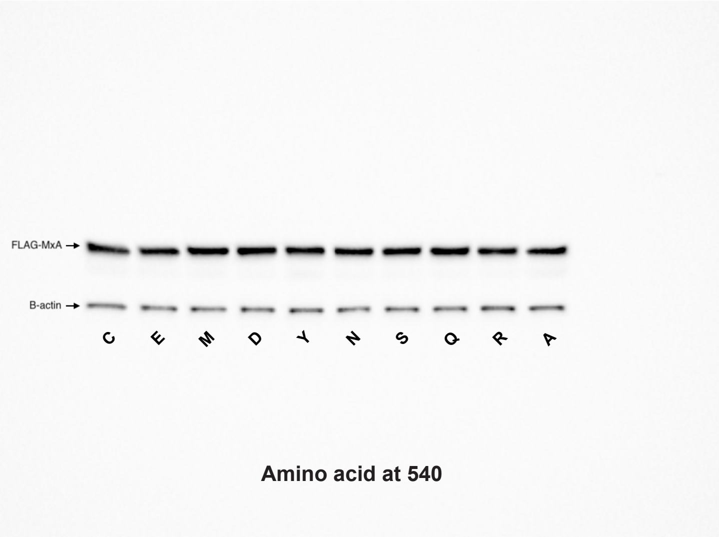

Fig 4B

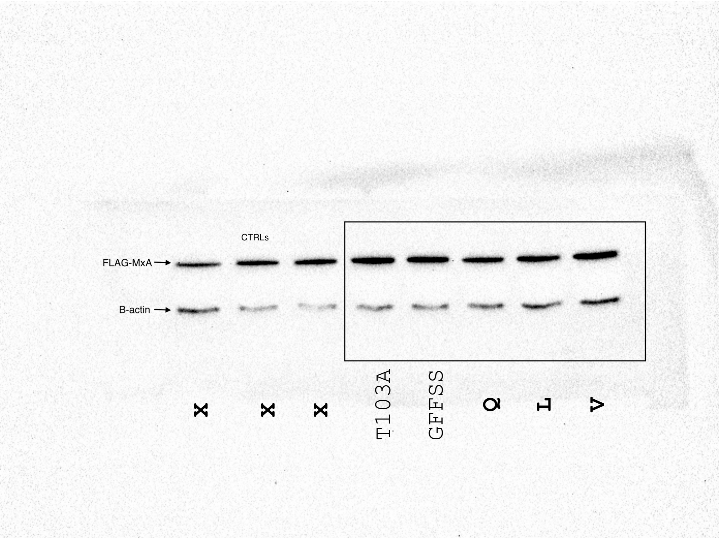

Fig 4C

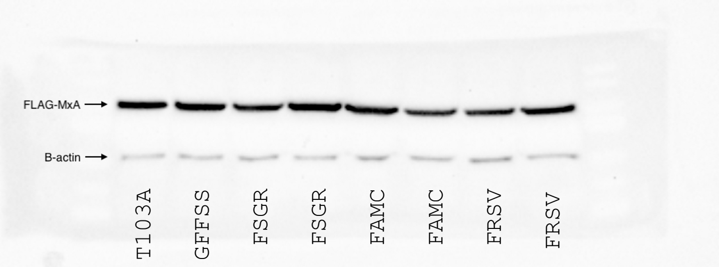

Fig 4D (top row)

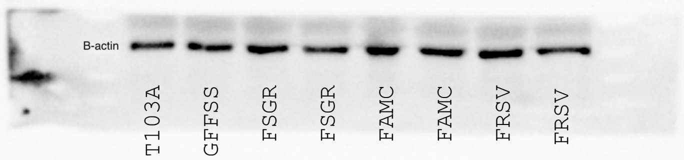

Fig 4D (bottom row)

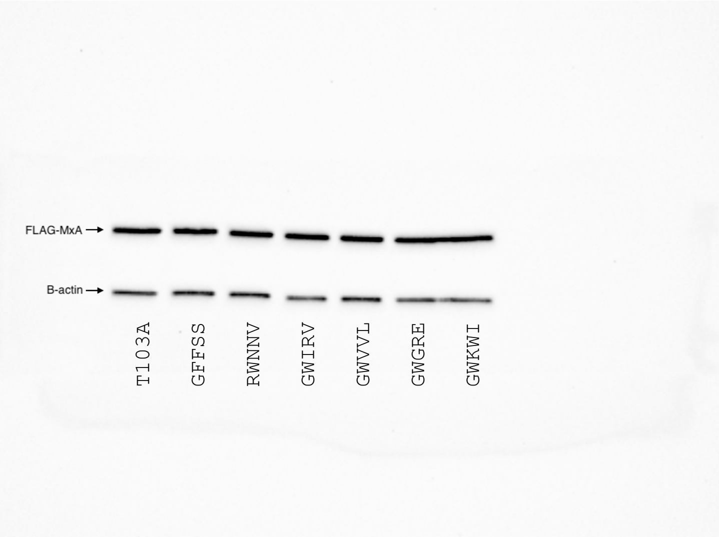

Fig 5A

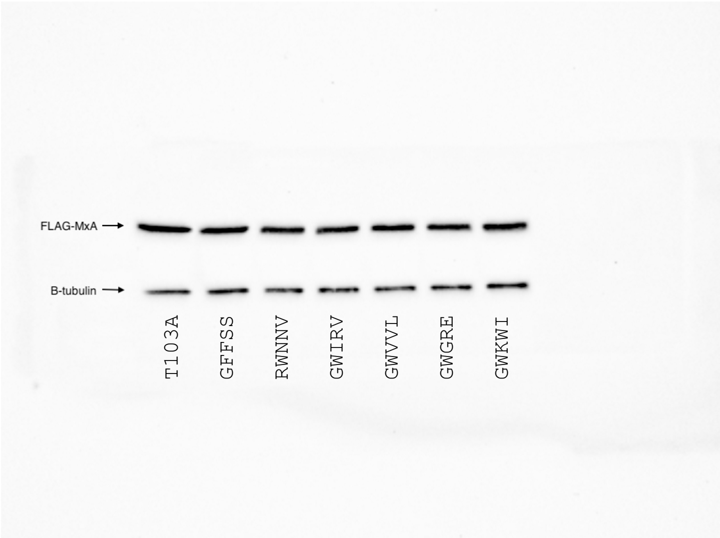
FLAG-MxA
B-tubulin
T103A
GFFSS
RWNNV
GWIRV
GWVVL
GWGRE
GWKWI

Fig 5B
